# Supplementary material for: Dual self-assembly of supramolecular peptide nanotubes to provide stabilisation in water
Source: Nat Commun. 2019 Oct 17;10:4708. doi: 10.1038/s41467-019-12586-8 (PMC6797743; doi:10.1038/s41467-019-12586-8)
Supplement: Supplementary file 1 — Supplementary Information [file 41467_2019_12586_MOESM1_ESM.pdf]

Supporting Information

Dual self-assembly of supramolecular peptide nanotubes to provide stabilisation in water

Julia Y. Rho[†], Henry Cox[‡], Edward Mansfield[†], Sean H. Ellacott[†], Raoul Peltier[†], Johannes C. Brendel[†], Matthias Hartlieb[†], Thomas A. Waigh[‡§] and Sébastien Perrier[†‖⊥] *

[†] Department of Chemistry, University of Warwick, Coventry CV4 7AL, United Kingdom.

[‡] Biological Physics, School of Physics and Astronomy, University of Manchester, Manchester M13 9PL, United Kingdom

[§] Photon Science Institute, University of Manchester, Manchester M13 9PL, United Kingdom

[‖] Faculty of Pharmacy and Pharmaceutical Sciences, Monash University, Parkville VIC 3052, Australia

[⊥] Warwick Medical School, University of Warwick, Coventry CV4 7AL, U.K.

Corresponding author: * S.perrier@warwick.ac.uk

Sample No.	Compound [a]	ESI (g mol^{-1})		
		**Calculated**	**Found**	**Attribution**
**9**	LP - standard	1499.7	1499.9	$[M+H]^{+}$
**10**	CP - standard	1081.7	1081.7	$[M+H]^{+}$
**11**	CP-Dde protected	1509.9	1509.94	$[M+Na]^{+}$
**12**	CP-Dde deprotected	1323.9	1323.8	$[M+H]^{+}$
**13**	Cy3-CP-Boc protected	892.6	892.9	$[M+Na]^{2+}$
**14**	Cy3-CP-Boc deprotected	731.5	731.8	$[M+H]^{2+}$
**15**	Cy5-CP-Boc protected	905.6	905.9	$[M+Na]^{2+}$
**16**	Cy5-CP-Boc deprotected	744.5	744.8	$[M+H]^{2+}$

Supplementary Table 1. Electrospray ionisation (ESI) - Mass spectrometry (MS) characterisation of cyclic peptide and dye conjugates carried out on the Agilent 6130B single Quad

[a] LP = linear peptide, CP = cyclic peptide.

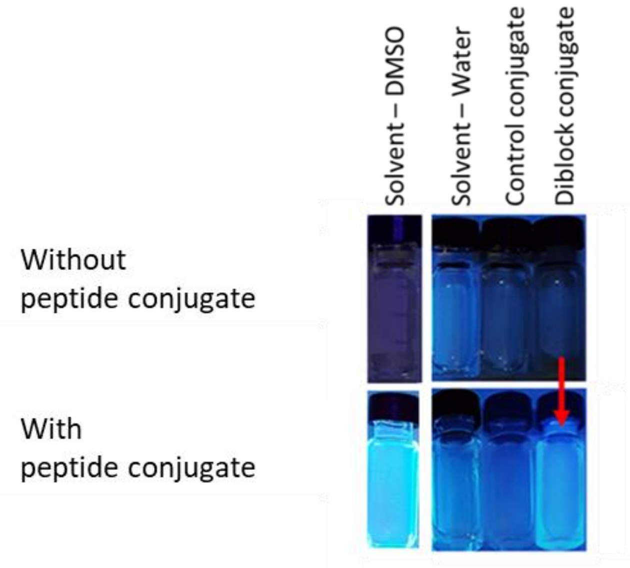

Supplementary Figure 1. Images of cyclic peptide polymer conjugates with the addition of DPH dye. Upon dye addition, fluorescence was observed in the case of the diblock conjugate solution. No visible fluorescence was observed for the control conjugate, absence of the hydrophobic core.

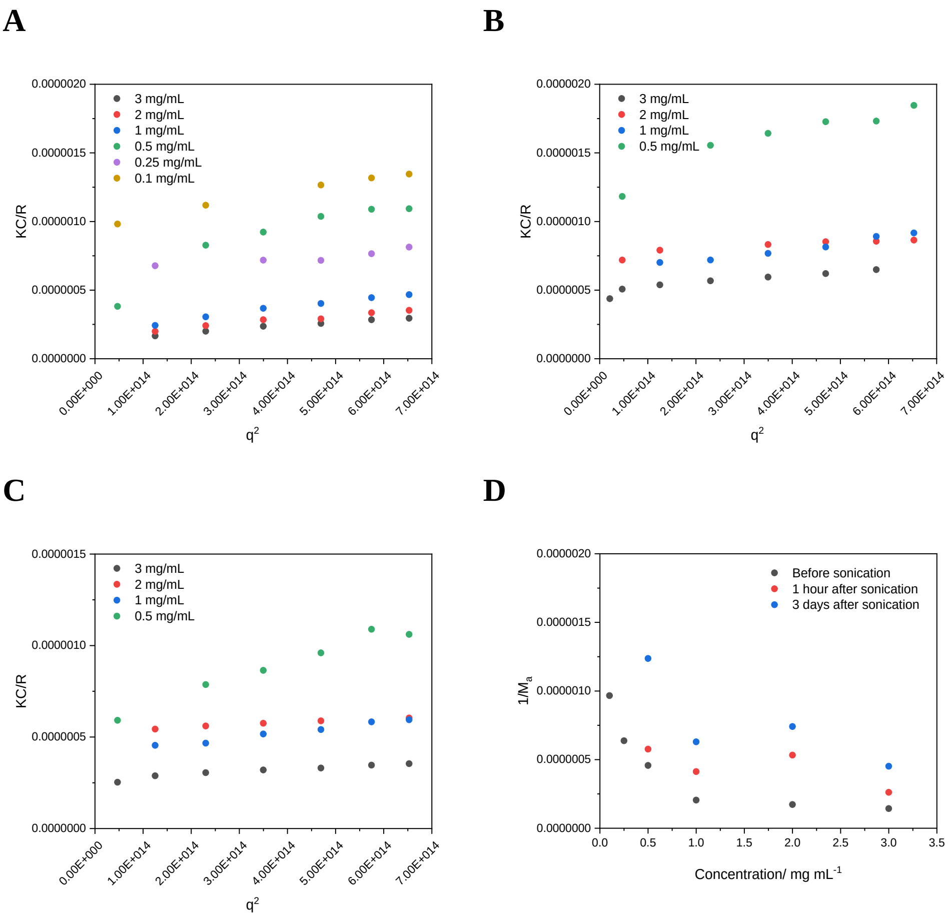

Supplementary Figure 2. Evolution of KC/R of diblock conjugate (CP-(pBA-pDMA)$_2$) in water as a function of q^2 obtained by static light scattering. A) Before sonication, B) 1 hour after sonication and C) 3 days after sonication. D) Evolution of 1/M_a of diblock conjugate in water as a function of concentration before sonication, 1 hour after and 3 days after sonication obtained by static light scattering.

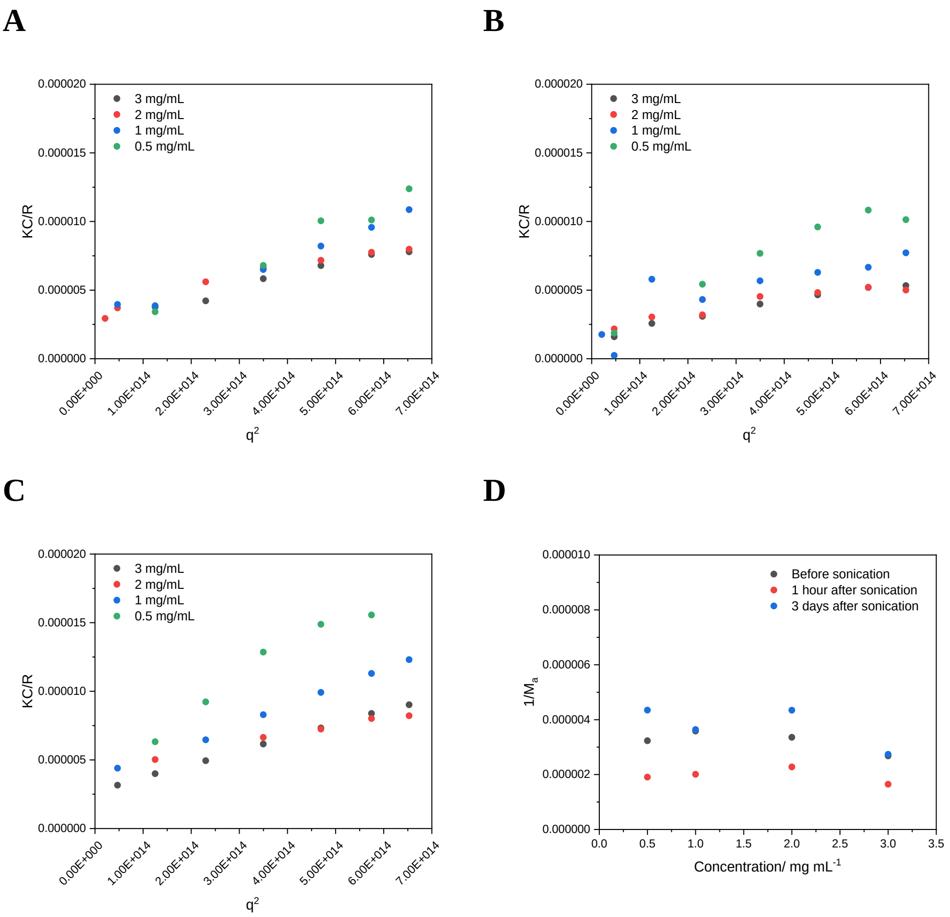

Supplementary Figure 3. Evolution of KC/R of control conjugate (CP-(pDMA)$_2$) in water as a function of q^2 obtained by static light scattering. A) Before sonication, B) 1 hour after sonication and C) 3 days after sonication. D) Evolution of $1/M_a$ of control conjugate in water as a function of concentration before sonication, 1 hour after and 3 days after sonication obtained by static light scattering.

	Before Sonication				1 Hour After Sonication				3 Days After Sonication			
Conc/ mg ml^{-1}	Control conjugate		Diblock conjugate		Control conjugate		Diblock conjugate		Control conjugate		Diblock conjugate	
	N_{agg}	Length/nm	N_{agg}	Length/nm	N_{agg}	Length/nm	N_{agg}	Length/nm	N_{agg}	Length/nm	N_{agg}	Length/nm
3.0	30	14	830	390	32	15	314	148	29	13	263	124
2.0	24	11	690	324	23	11	154	72	19	9	161	76
1.0	22	10	581	273	27	13	199	94	22	10	189	89
0.5	25	12	260	122	28	13	95	45	19	9	96	45
0.25	23	11	187	88	-	-	-	-	-	-	-	-
0.10	-	-	123	58	-	-	-	-	-	-	-	-
Average	25	11	-	-	28	14	-	-	22	10	-	-

Supplementary Table 2. Table of calculated length of the nanotubes from static light scattering over a range of concentrations (0.1 – 3.0 mg mL^{-1}) before and after sonication.

Supplementary Discussion. The following discussion includes the SANS scattering data, models and fitting parameters for the diblock and control conjugates. In the case of the diblock conjugate, a clear fit to the hairy cylinder is shown and for the control conjugate a comparison between the hairy cylinder and sphere models have been plotted and discussed.

V_{brush} - the molecular volume of the polymer arms - *Calculated as the M_w of the polymer divided by Avogadro's constant (N_a) and multiplied by the density*

R_core = 5 Å fixed as the radius of the CP

d – fixed at 1 – non penetration of chains into the cylindrical core of the CP

eta_core, eta_brush, eta_solv – SLD value calculated using molecular structures

Diblock conjugate – Contribution 1 – CYL+Chains (RW)

**Parameters**	**Fit**	**Value**	**Units**
Delta	✓	2.45747e-5	
R_core		5	Å
n_agg	✓	0.0601087	
V_brush		14776	cm^3
eta_core		8.20995e-007	$Å^{-2}$
eta_brush		1.64e-006	$Å^{-2}$
eta_solv		6.33e-006	$Å^{-2}$
xsolv_core	✓	183.893	
Rg	✓	13.9993	Å
h		1	
H	✓	1000	Å

Diblock conjugate - Contribution 2 – extended Guinier law

**Parameters**	**Fit**	**Value**	**Units**
Delta	✓	0.133184	
I0	✓	0.133184	
a	✓	0.88974	
Ra	✓	41.9261	

chisqr: 256, red. chisqr: 6.57

Control conjugate - CYL+Chains (RW) - chisqr: 26.8, red. chisqr: 1.03

**Parameters**	**Fit**	**Value**	**Units**
Delta	✓	0.083845	
R_core		5	Å
n_agg	✓	0.00453034	
V_brush		15757	cm^3
eta_core		8.20995e-007	$Å^{-2}$
eta_brush		9.57751e-007	$Å^{-2}$
eta_solv		6.33e-006	$Å^{-2}$
xsolv_core	✓	0.572701	
Rg	✓	12.0219	Å
h		1	
H	✓	492.444	Å

Control conjugate - Sphere+Chains (RW) - chisqr: 1439.13, red. chisqr: 29.98

**Parameters**	**Fit**	**Value**	**Units**
Delta	✓	134.251	
R_core		5	Å
n_agg	✓	0.00500811	
V_brush		15757	cm^3
eta_core		8.20995e-007	$Å^{-2}$
eta_brush		9.57751e-007	$Å^{-2}$
eta_solv		6.33e-006	$Å^{-2}$
xsolv_core	✓	0.57	
Rg	✓	76.5148	Å
d		1	

Here for comparison, the spherical and elongated (cylindrical) micelles models have been fitted for the control conjugate (**4**). From the data we can observe the control conjugates fit best to an elongated micelle structure – see above for chi values. Particularly of note is the q dependence in the Guinier region best fits the elongated (hairy cylinder) model.

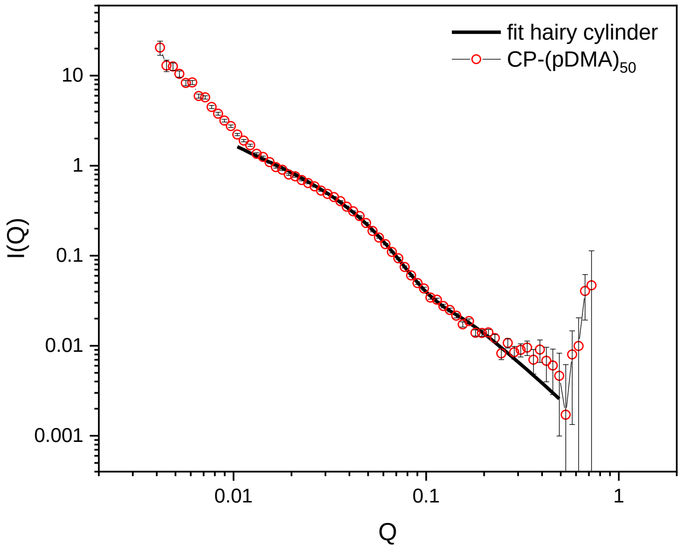

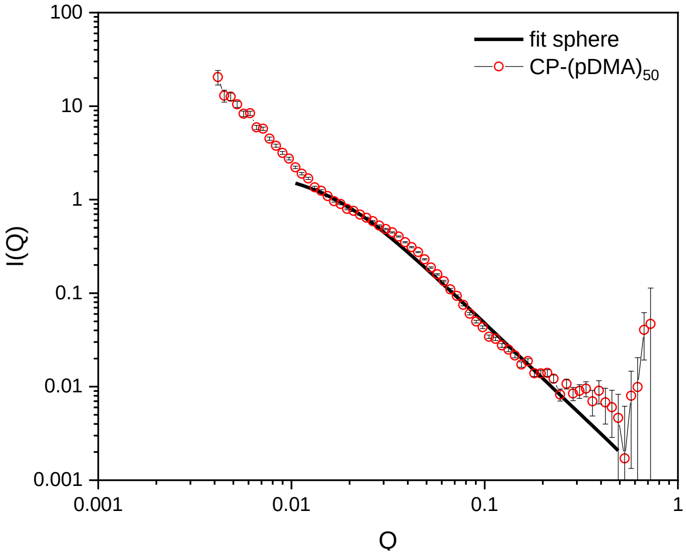

Magnification - 15,000

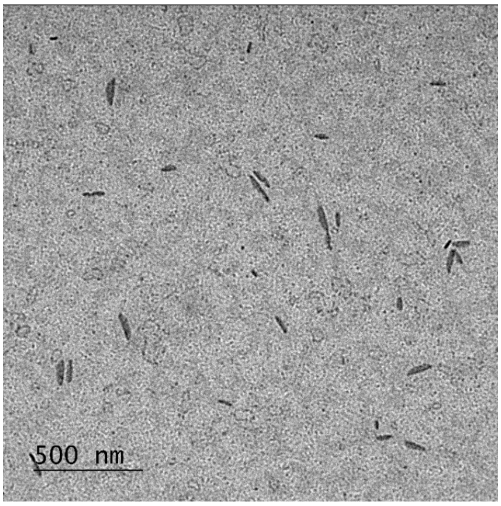

Magnification - 15,000

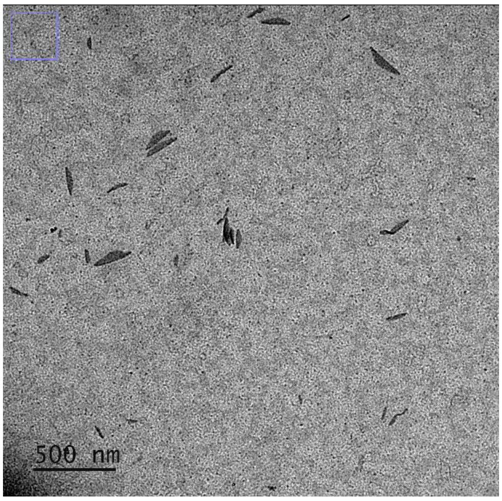

Magnification - 40,000

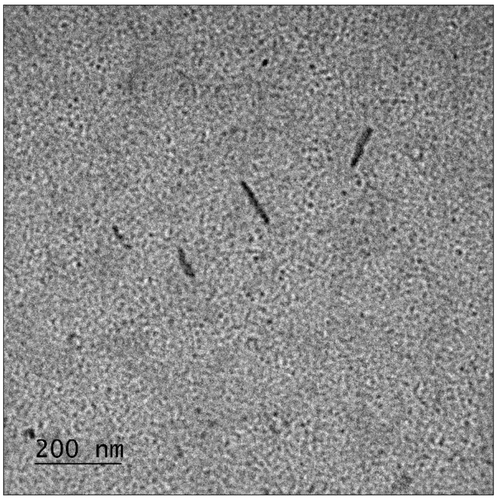

Magnification - 25,000

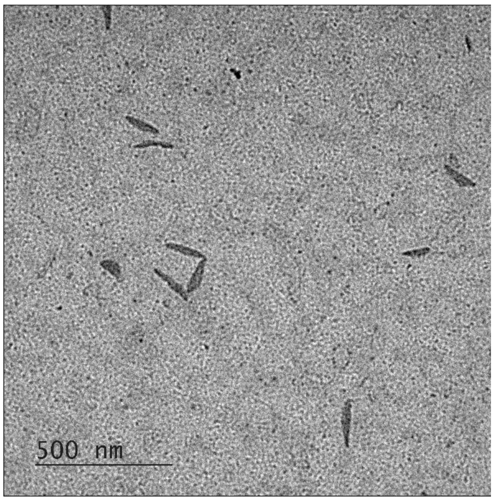

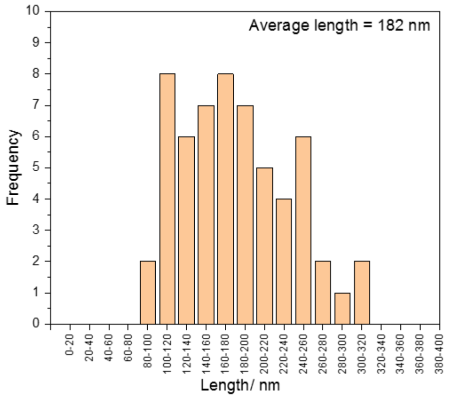

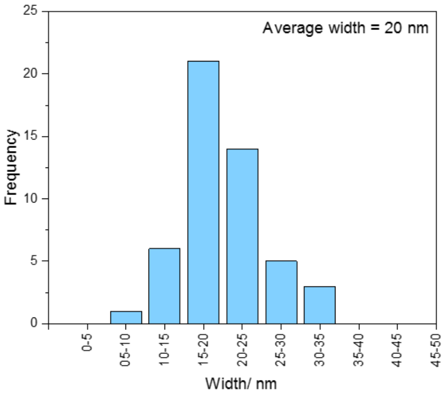

Supplementary Figure 4. TEM images of diblock conjugated cyclic peptides (CP-(pBA-pDMA)$_2$) prepared in water (1 mg/mL).

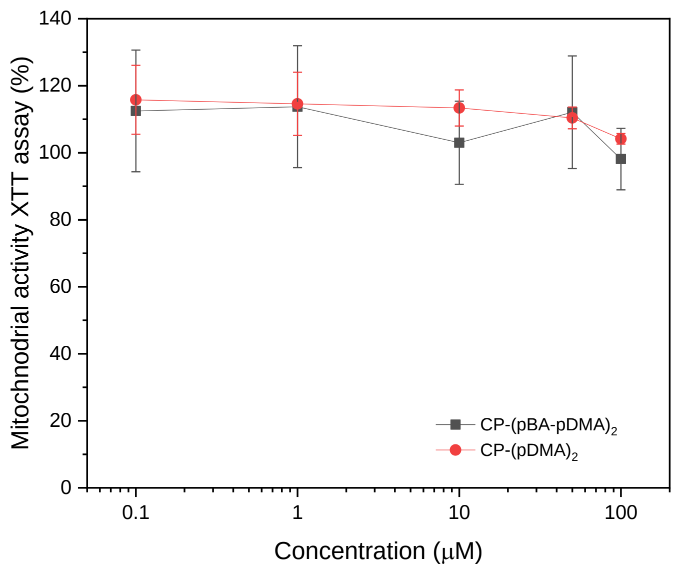

Supplementary Figure 5. Toxicity profile for diblock (CP-(pBA-pDMA)$_2$) and control (CP-(pDMA)$_2$) conjugates.

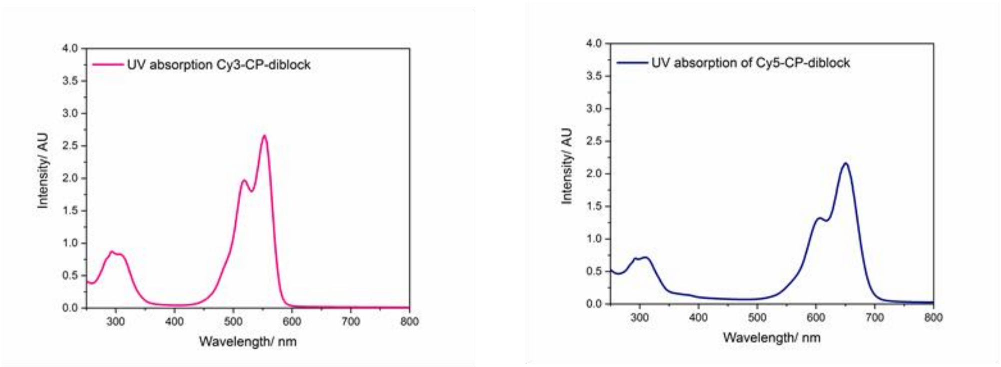

Excitation $_{max}$ = 554 nm   Excitation $_{max}$ = 652 nm

Supplementary Figure 6. UV absorption spectra of Cy3-CP-Diblock and Cy5-CP-Diblock conjugates in water at 35 µM.

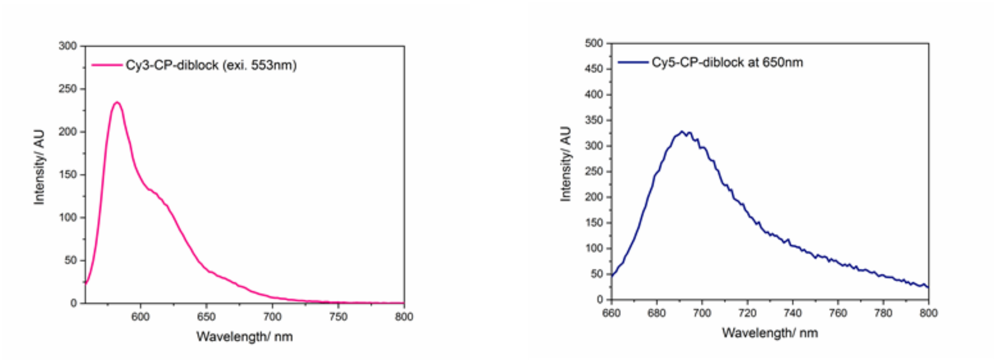

Emission $_{max}$ = 582 nm   Emission $_{max}$ = 692 nm*

***N.B** Cy5 conjugate notably less fluorescent (Detector voltage set to high 800 V)

Supplementary Figure 7. Fluorescence emission spectra of Cy3-CP-Diblock and Cy5-CP-Diblock conjugates in water at 35 µM.

A Cy3-CP-(pBA-pDMA)$_2$
and Cy5-CP-(pBA-pDMA)$_2$

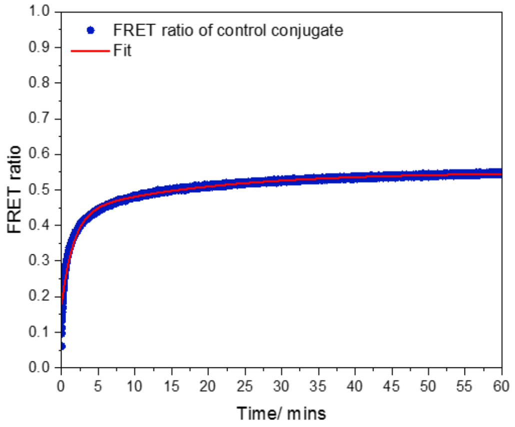

B Cy3-CP-(pDMA)$_2$
and Cy5-CP-(pDMA)$_2$

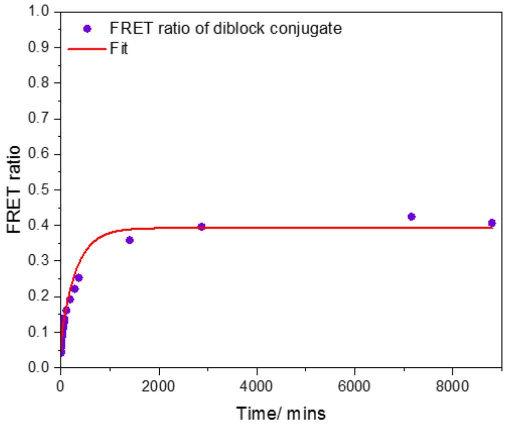

Model	ExpDec2
Equation	y = A1*exp(-x/t1) + A2*exp(-x/t2) + y0
Plot	B
y0	0.54954 ± 6.0585E-4
A1	-0.25226 ± 0.00167
t1	1.63271 ± 0.01406
A2	-0.11525 ± 9.98507E-4
t2	18.85467 ± 0.41719
Reduced Chi-Sqr	1.0082E-4
R-Square(COD)	0.98248
Adj. R-Square	0.98247

Model	ExpDec2
Equation	y = A1*exp(-x/t1) + A2*exp(-x/t2) + y0
Plot	B
y0	0.39297 ± 0.00118
A1	-0.02821 ± 2.11023E-4
t1	5.76864 ± 0.08967
A2	-0.30887 ± 0.00117
t2	321.12536 ± 2.00909
Reduced Chi-Sqr	5.46154E-6
R-Square(COD)	0.98931
Adj. R-Square	0.9893

Supplementary Figure 8. The change in FRET ratio over time upon mixing of a) Cy3 and Cy5 diblock conjugates, b) Cy3 and Cy5 control conjugates.

Supplementary Methods: Particle tracking and Stochastic Optical Reconstruction Microscopy (STORM) experiments were performed with the sample at 1 mg/ml peptide concentration dissolved in de-ionised water (>18 MΩ) and then filtered using a 0.45 μm cellulose acetate syringe filter (SupaTop). Images of the peptide aggregates were captured on our specially constructed STORM microscope and full details of the microscope is detailed in our previous work.[7] For both experiments circular imaging spacers (Thermo Scientific) were fixed to cleaned glass microscope slides to create wells for the sample. Then, for the particle tracking experiments, 7.5 μL of the peptide solution was deposited in the well formed by the spacer and the slide and a circular glass coverslip was used to seal the sample into the well. The sample was illuminated and the diffusion of aggregates through the solvent was videoed at 100 fps for 30 secs. Care was taken to ensure only aggregates at least 10 μm from the surface were recorded to eliminate the risk of hydrodynamic drag from the surfaces. Background subtraction was performed in ImageJ on the raw videos using the "rolling-ball" algorithm.[8] The position of particles in the processed videos were then tracked using our previously developed particle tracking software.[9]

For the STORM experiments, 3 μL of sample was placed directly onto a cleaned glass coverslip to allow adsorption of the aggregates to the glass. Then, 4 μL of OxEA STORM imaging buffer[10] was placed into the well on the microscope slide. The coverslip was then fixed over the well such that the imaging buffer and sample came into contact with each other and the total volume of liquid in the well was 7 μL. For the STORM images, at least 5,000 images of each aggregate were recorded at high laser intensity. To capture two colour images, two raw data sets were captured sequentially for each STORM image, one for the Cy5 dye (647 nm laser) and then another for the Cy3 dye (568 nm laser). Localisation of fluorophores in the raw data was then performed with the ImageJ plugin ThunderSTORM.[11] The two resulting single colour images (one for Cy5 dye and one for Cy3 dye) were then combined into a two colour image. We calculated and corrected for any drift of the sample between recording the two single colour images by calculating the 2D cross-correlation of the Cy3 and Cy5 images. The peak of the 2D cross-correlation gave us an estimation of the drift and this was typically between 0 and 50 nm, we could then translate either the Cy3 or Cy5 image and recombine them to form the final two colour image with correct alignment between the colour channels.

To find the hydrodynamic radius of aggregates in the particle tracking experiments we calculated the ensemble averaged mean square displacement (MSD) of all the tracks output from our particle tracking software. In a purely viscous fluid, like the water used here, the MSD is linearly dependent upon time or lag time, τ, and

$$\langle r \rangle = 2nD\tau^{\alpha}, \quad (4)$$

where n is the number of dimensions, D is the diffusion co-efficient and α is the power law exponent (α=1 for purely viscous fluids).[12] To verify our data, we performed a power law fit to Equation (4) and the results are shown in Supplementary Figure 9. In all cases the MSD was found to be linearly dependent on lag time with power law exponents of 1.0-1.1 in all cases, as expected. Using the gradient of each fit, the hydrodynamic radius, r_h, was found using the relation between it and the diffusion co-efficient,

$$D = \frac{kT}{6\pi\eta r_h}, \quad (5)$$

where η is the viscosity of water and kT is the thermal energy.[12] Particle tracking experiments were performed individually on the Cy3 and Cy5 labelled peptides as well as mixtures of the Cy3 and Cy5 peptides when mixed before assembly (Premixed) or when assembled and then mixed in solution (after co-injection). All the results were similar with a hydrodynamic radius of approximately 50 nm, except for the aggregates formed by just Cy5 labelled peptides which were slightly smaller at 33 ± 1 nm, as shown in Supplementary table 4.

Supplementary Table 4. Tabular results of the particle tracking experiments. The fit was performed on the ensemble averaged MSD which was calculated from the average of many MSDs calculated from each individual track and the number in each sample is greater than 1,000 as shown. The power law fit exponent is shown and is close to 1 as expected. A control experiment was also performed using fluorescent beads with a radius of 50 nm. Our results agreed with the manufactures specification of the size.

Sample	Number of tracks	Power law fit exponent	Hydrodynamic radius (nm)
Cy3-CP-Diblock	1704	1.04	52 ± 2
Cy5-CP-Diblock	1100	1.06	33 ± 1
1 day after co-injection	1996	1.03	50 ± 2
30 day after co-injection	2434	1.06	46 ± 2
Premixed	3480	1.06	49 ± 2
50 nm beads	18130	1.05	49 ± 2

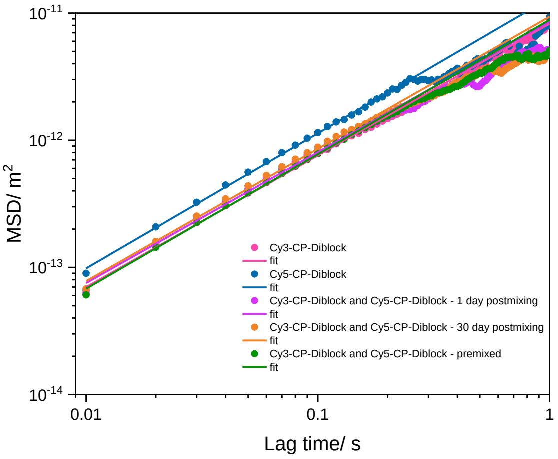

Supplementary Figure 9. The MSD as a function of lag time for the experimental data (filled circles) and the fit to each data set (solid line). The Cy3-CP-Diblock, co-injected Cy3-CP-Diblock and Cy5-CP-Diblock conjugate and premixed samples all have similar hydrodynamic radii of about 50 nm and the Cy5 aggregates are slightly smaller with a hydrodynamic radius of 33 ± 1 nm. All MSDs were linearly dependent on lag time showing that the particles exhibited the expected Brownian motion and that the data was of high quality. Each MSD shown is the ensemble average from at least 1,000 individual particle MSDs.

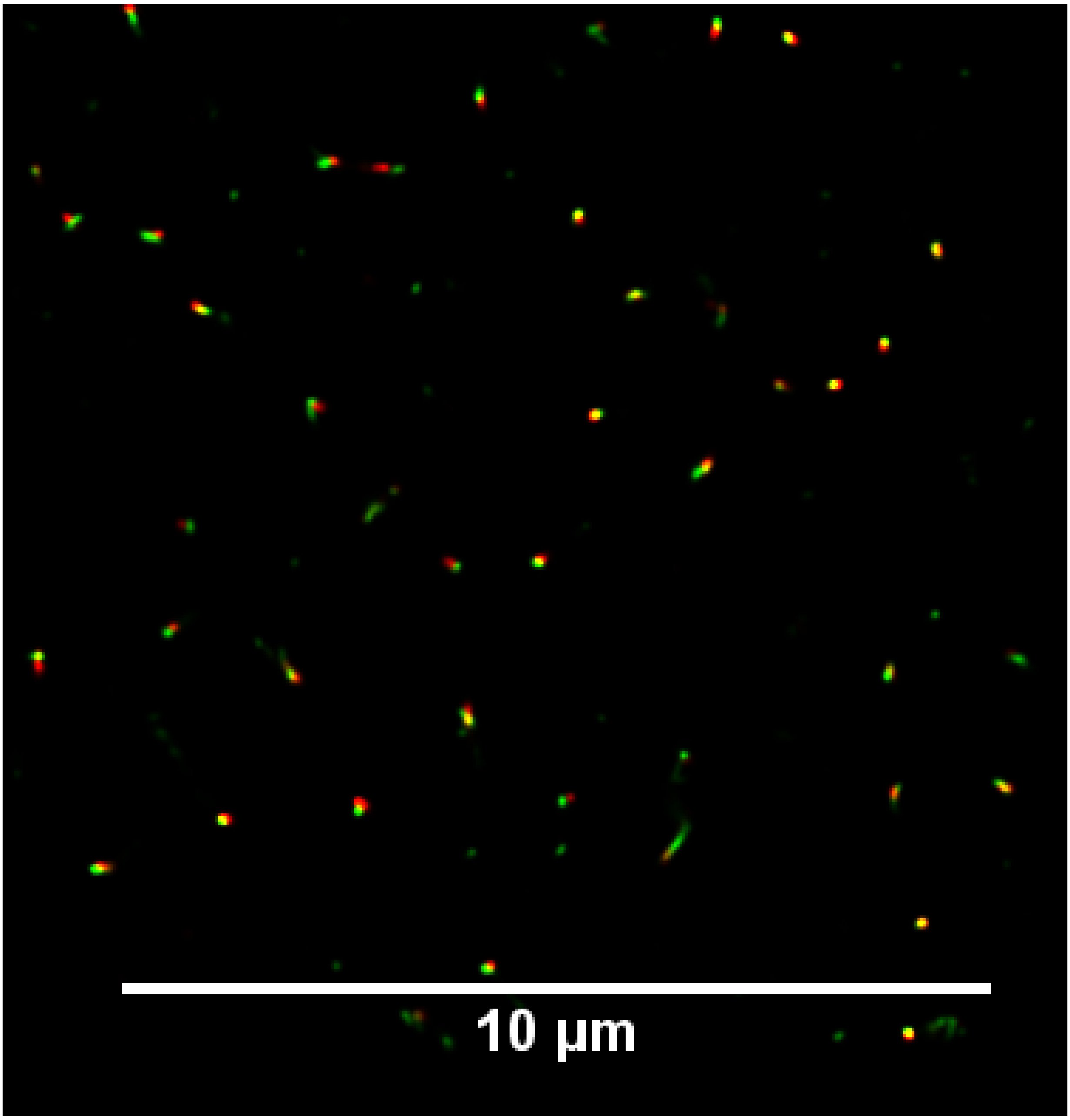

Supplementary Figure 10. A STORM image of the premixed sample, the scale bar is 10 µm (left). This image further shows that almost all aggregates feature some degree of co-localisation and are therefore formed of a mixture of Cy3 and Cy5 labelled peptides.

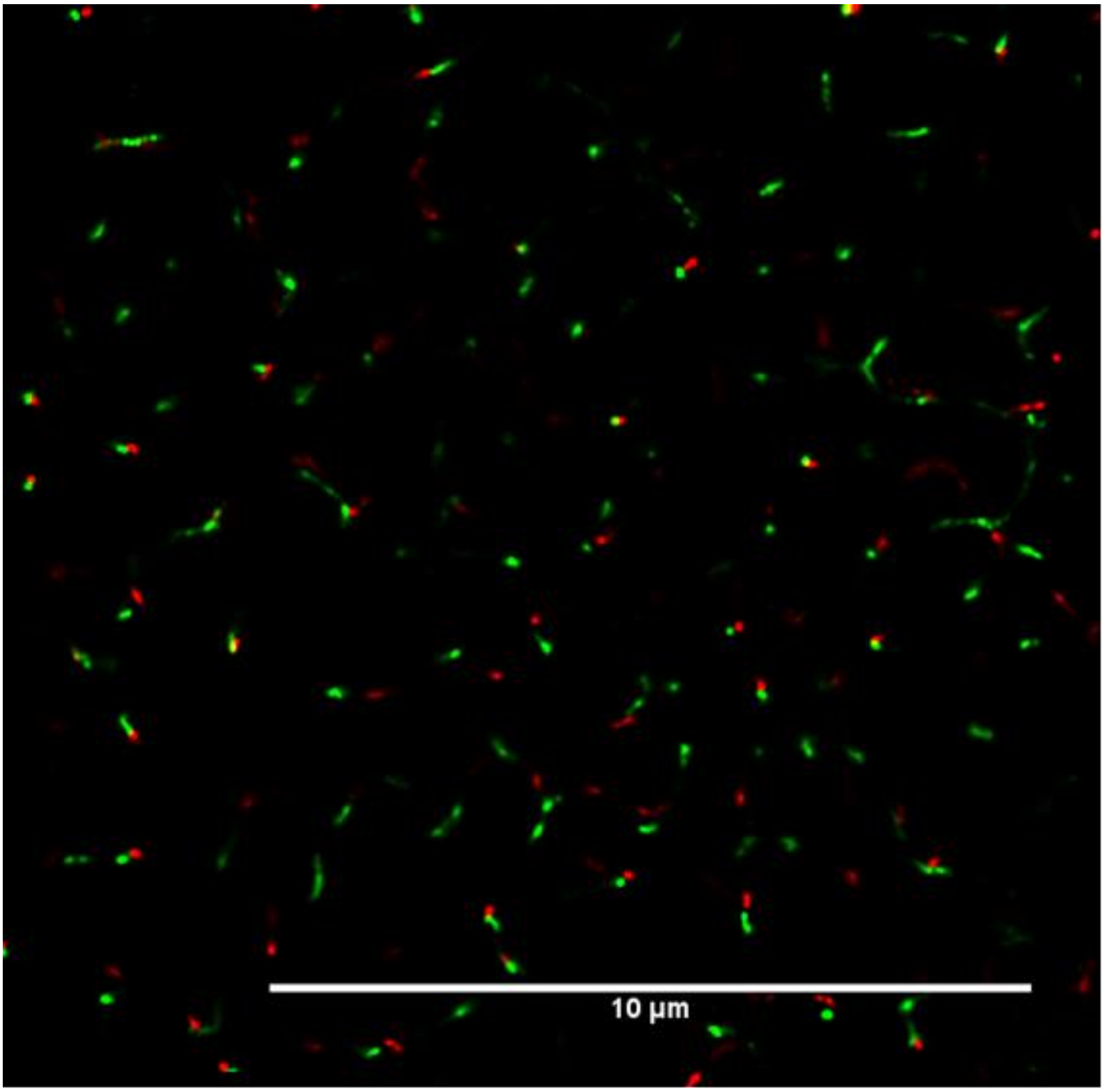

Supplementary Figure 11. A STORM image of the sample 1 day after co-injection of Cy3 and Cy5 diblock conjugate, the scale bar is 5 µm long. After one day many of the aggregates are co-localised in the co-injected sample, however not to the same degree as in Figure S9, S10 or Figure 8 (bottom) of the manuscript. However, you can see how some of the aggregates have attached to each other at the ends, or potentially formed new multi-coloured aggregates since mixing.

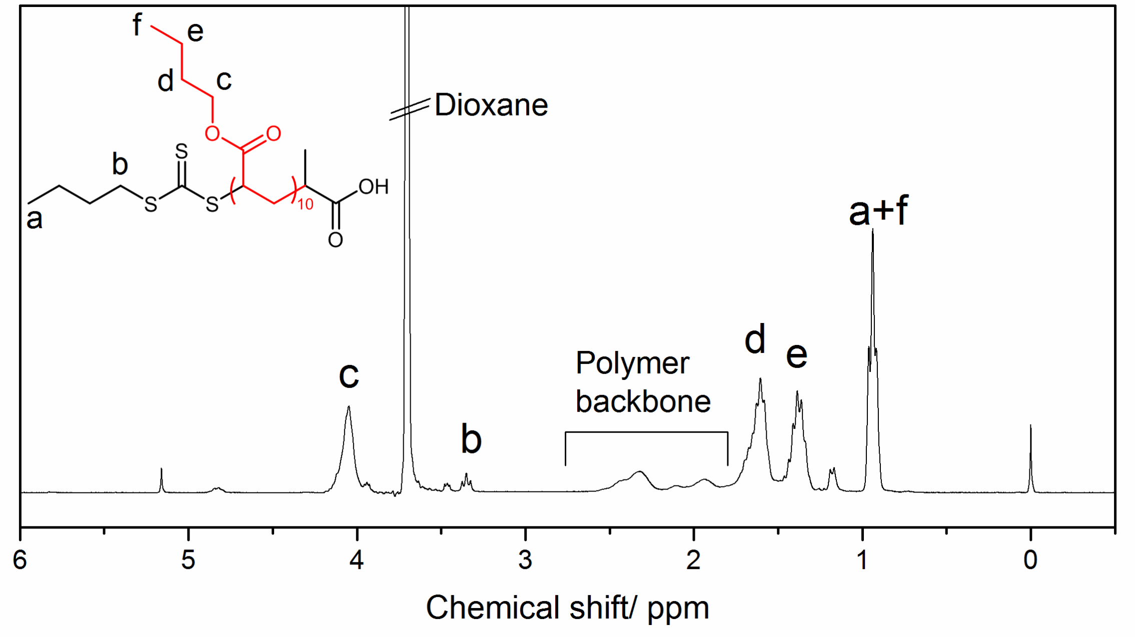

Supplementary Figure 12. ^{1}H NMR of pBA (17) polymer

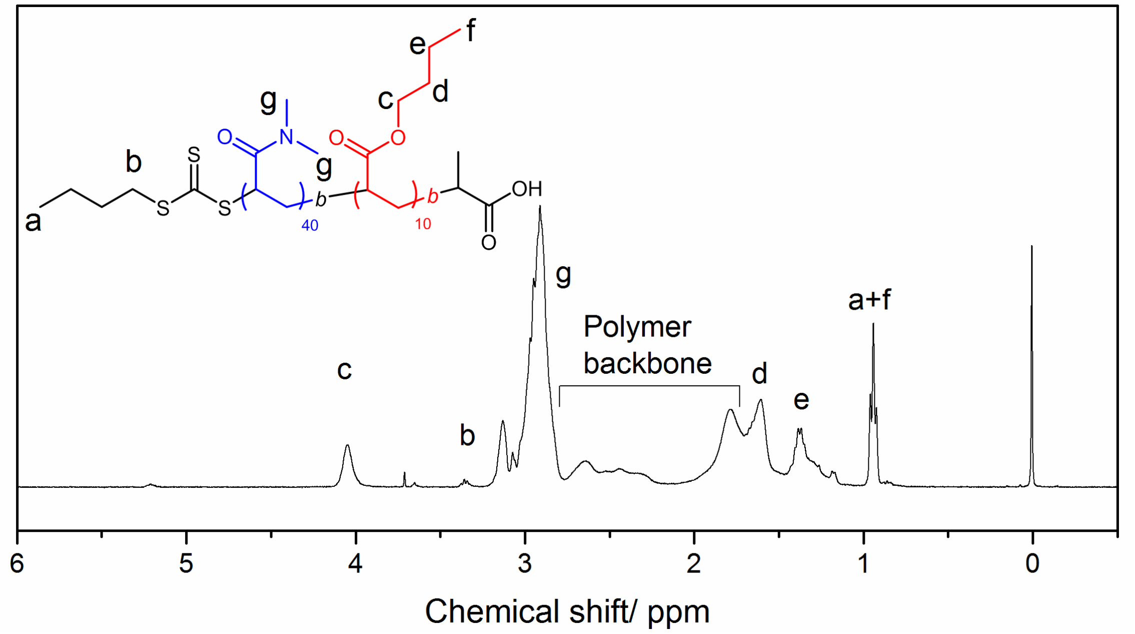

Supplementary Figure 13. ^{1}H NMR of pBA-pDMA (1) polymer

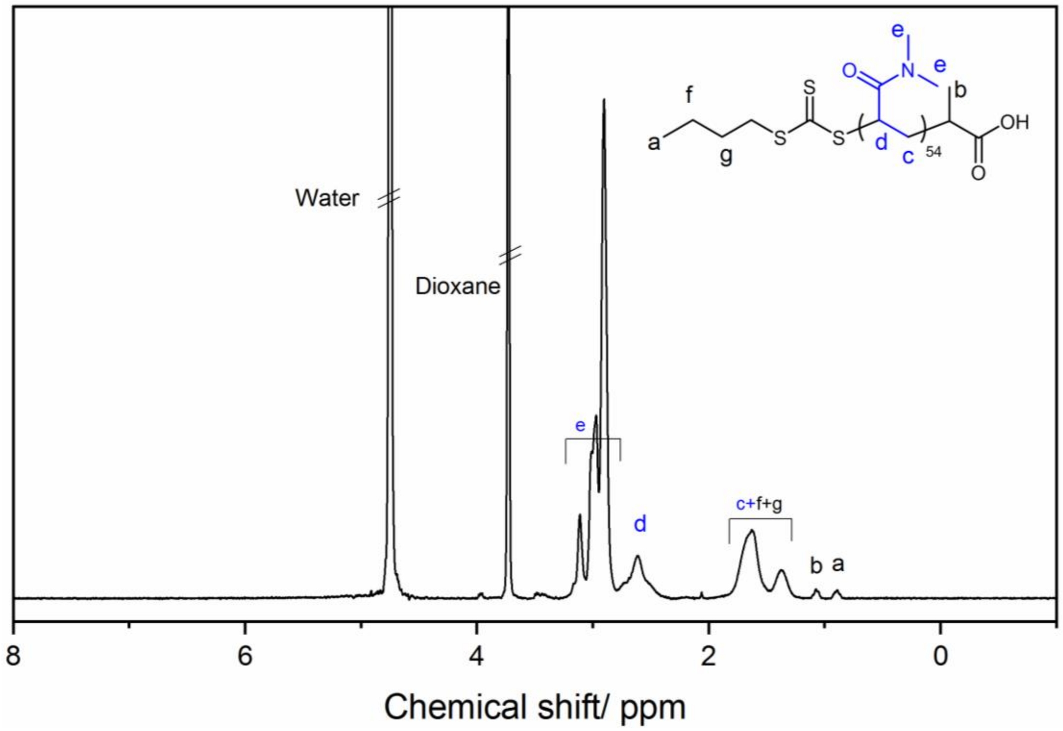

Supplementary Figure 14. ^{1}H NMR of pDMA (2)

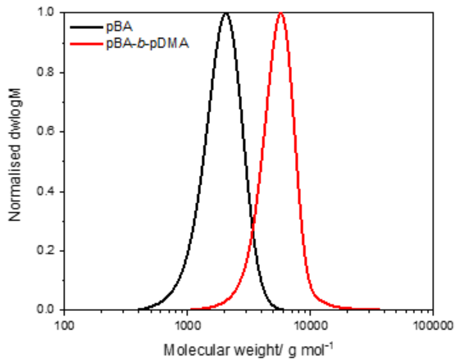

pBA
$Đ$ = 1.15
$M_{w,GPC}$= 2,000 g mol^{-1}
$M_{w,NMR}$= 1,520 g mol^{-1}

pBA-*b*-pDMA
$Đ$ = 1.13
$M_{w,GPC}$= 5,700 g mol^{-1}
$M_{w,NMR}$= 5,590g mol^{-1}

Supplementary Figure 15. Size exclusion chromatograms of pBA and pBA-pDMA (from THF SEC, DRI detector, PMMA and PS standard).

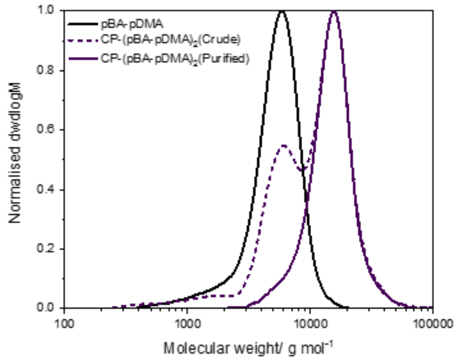

pBA-*b*-pDMA
$Đ$ = 1.34
$M_{w,GPC}$= 5,600 g mol^{-1}

CP-(pBA-*b*-pDMA)$_2$
$Đ$ = 1.21
$M_{w,GPC}$= 15,800 g mol^{-1}
$M_{w,\ Theory}$= 12,200 g mol^{-1}

Supplementary Figure 16. Size exclusion chromatograms of diblock polymer (pBA-pDMA) and diblock conjugate (CP-(pBA-pDMA)$_2$) before and after purification (from DMF + 0.1% LiBr SEC, DRI detector, PMMA standard). The removal of the polymer can be followed by the disappearance of the low molecular weight distribution. Also in main manuscript as Figure 2.

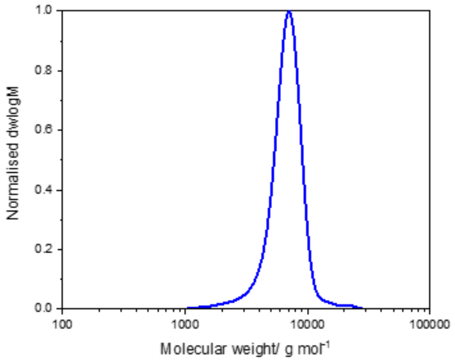

pDMA
$Đ$ = 1.14
$M_{w,GPC}$= 7,000 g mol^{-1}
$M_{w,NMR}$= 5,690 g mol^{-1}

Supplementary Figure 17. Size exclusion chromatogram of (from DMF + NH4BF4 additive eluent, SEC, DRI detector, PMMA standard) of the homopolymer pDMA.

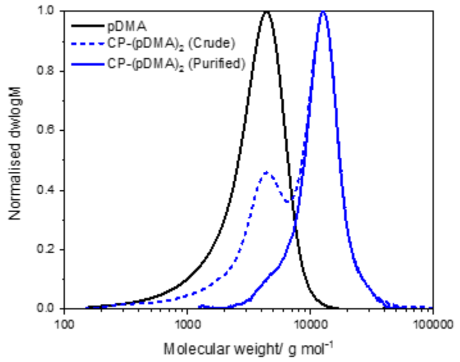

pDMA
$Đ$ = 1.58
$M_{w,GPC}$= 4,400 g mol^{-1}

CP-(pDMA)$_2$
$Đ$ = 1.21
$M_{w,GPC}$= 13,000 g mol^{-1}

Supplementary Figure 18. Size exclusion chromatograms of diblock polymer (pDMA) and diblock conjugate (CP-(pDMA)$_2$) before and after purification (from DMF + 0.1% LiBr SEC, DRI detector, PMMA standard). Also in main manuscript as Figure 2.

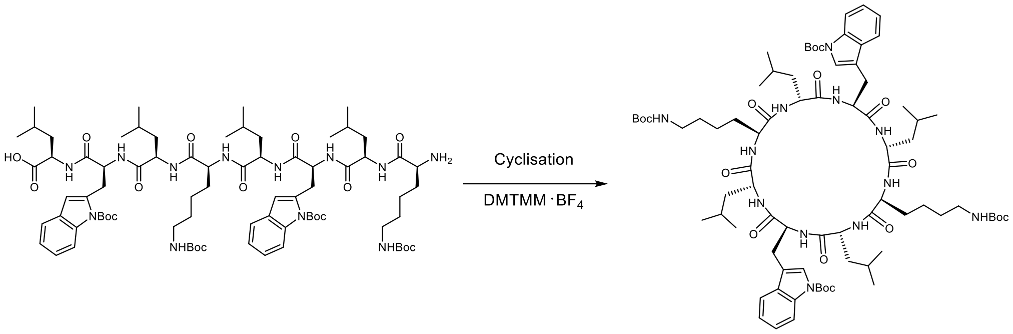

Supplementary Figure 19. Preparation of the linear (9) and cyclic peptide.[1]

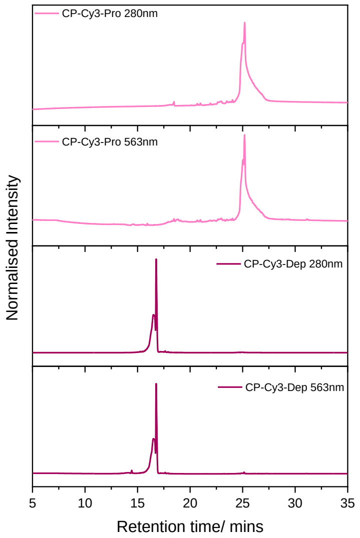

Supplementary Figure 20. High Performance Liquid Chromatography (HPLC) spectra of the CP-Cy3-Protected (13) and CP-Cy3-Deprotected (14). Detector set to 280 nm (tryptophan of cyclic peptide) and 563 nm (Cyanine 3 emission).

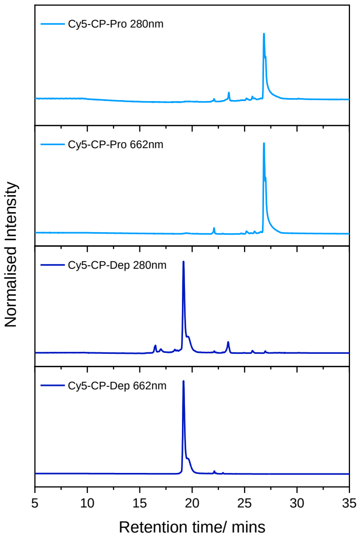

Supplementary Figure 21. High Performance Liquid Chromatography (HPLC) spectra of the Cy5-CP-Pro (15) and CP-Cy5-Deprotected (16). Detector set to 280 nm (tryptophan of cyclic peptide) and 662 nm (Cyanine 5 emission).

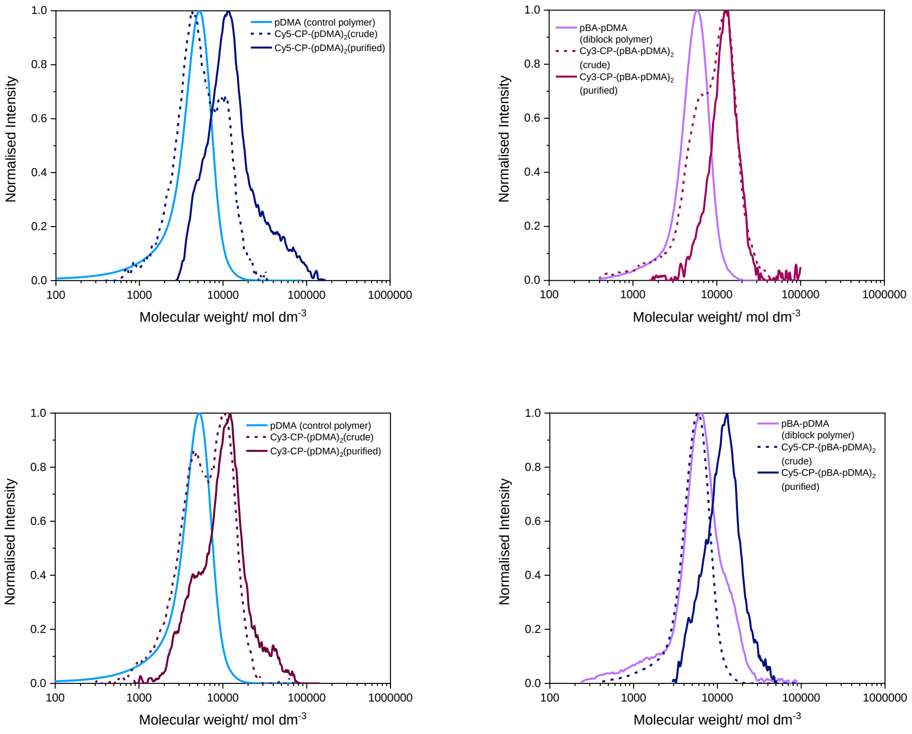

N.B. Due to the amounts and low yields obtained for these compounds, very dilute samples were measured and therefore a high signal to noise was observed.

Supplementary Figure 22. Size exclusion chromatography (SEC) of dye conjugates (from DMF + 0.1% LiBr SEC, DRI detector, PMMA standard).

Supplementary References

1. Larnaudie, S.C., Brendel, J.C., Jolliffe, K.A. & Perrier, S. Cyclic peptide–polymer conjugates: Grafting-to vs grafting-from. *J. Polym. Sci., Part A: Polym. Chem.* **54,** 1003-1011 (2016).
2. Shaikh, H. *et al.* Hydrogel and Organogel Formation by Hierarchical Self-Assembly of Cyclic Peptides Nanotubes. *Chem. - Eur. J.* **24,** 19066-19074 (2018).
3. VARLEY, F.S. Neutron scattering lengths and cross sectioirn. *Neutron News* **3**(1992).
4. NIST, Available at https://www.ncnr.nist.gov/resources/n-lengths/.
5. Hendrikse, S.I.S. *et al.* Controlling and tuning the dynamic nature of supramolecular polymers in aqueous solutions. *Chem. Commun.* **53,** 2279-2282 (2017).
6. Rho, J.Y. *et al.* Probing the Dynamic Nature of Self-Assembling Cyclic Peptide–Polymer Nanotubes in Solution and in Mammalian Cells. *Adv. Funct. Mater.* **28,** 1704569 (2018).
7. Cox, H., Georgiades, P., Xu, H., Waigh, T.A. & Lu, J.R. Self-Assembly of Mesoscopic Peptide Surfactant Fibrils Investigated by STORM Super-Resolution Fluorescence Microscopy. *Biomacromolecules* **18,** 3481-3491 (2017).
8. Sternberg Biomedical Image Processing. *Computer* **16,** 22-34 (1983).
9. Salman, S.R., Thomas, A.W., Xiubo, Z. & Jian, R.L. Precise particle tracking against a complicated background: polynomial fitting with Gaussian weight. *Phys. Biol.* **4,** 220 (2007).
10. Nahidiazar, L., Agronskaia, A.V., Broertjes, J., van den Broek, B. & Jalink, K. Optimizing Imaging Conditions for Demanding Multi-Color Super Resolution Localization Microscopy. *PLOS ONE* **11,** e0158884 (2016).
11. Ovesný, M., Křížek, P., Borkovec, J., Švindrych, Z. & Hagen, G.M. ThunderSTORM: a comprehensive ImageJ plug-in for PALM and STORM data analysis and super-resolution imaging. *Adv. Bioinf.* **30,** 2389-2390 (2014).
12. J Bonales, L., Maestro, A., Rubio, R. & Ortega, F., *Microrheology of Complex Fluids*. (2011).
